# Histological Analysis and 3D Reconstruction of Winter Cereal Crowns Recovering from Freezing: A Unique Response in Oat (*Avena sativa* L.)

**DOI:** 10.1371/journal.pone.0053468

**Published:** 2013-01-14

**Authors:** David P. Livingston, Cynthia A. Henson, Tan D. Tuong, Mitchell L. Wise, Shyamalrau P. Tallury, Stanley H. Duke

**Affiliations:** 1 United States Department of Agriculture-Agricultural Research Service, Raleigh, North Carolina, United States of America; 2 Department of Crop Science, North Carolina State University, Raleigh, North Carolina, United States of America; 3 United States Department of Agriculture-Agricultural Research Service, Cereal Crops Research Unit, Madison, Wisconsin, United States of America; 4 Department of Agronomy, University of Wisconsin, Madison, Wisconsin, United States of America; Instituto de Biología Molecular y Celular de Plantas, Spain

## Abstract

The crown is the below ground portion of the stem of a grass which contains meristematic cells that give rise to new shoots and roots following winter. To better understand mechanisms of survival from freezing, a histological analysis was performed on rye, wheat, barley and oat plants that had been frozen, thawed and allowed to resume growth under controlled conditions. Extensive tissue disruption and abnormal cell structure was noticed in the center of the crown of all 4 species with relatively normal cells on the outside edge of the crown. A unique visual response was found in oat in the shape of a ring of cells that stained red with Safranin. A tetrazolium analysis indicated that tissues immediately inside this ring were dead and those outside were alive. Fluorescence microscopy revealed that the barrier fluoresced with excitation between 405 and 445 nm. Three dimensional reconstruction of a cross sectional series of images indicated that the red staining cells took on a somewhat spherical shape with regions of no staining where roots entered the crown. Characterizing changes in plants recovering from freezing will help determine the genetic basis for mechanisms involved in this important aspect of winter hardiness.

## Introduction

Fall planting allows winter cereals like rye (*Secale cereale* L.), wheat (*Triticum aestivum* L.), barley (*Hordeum vulgare* L.) and oats (*Avena sativa* L.) to be harvested earlier the following spring and generally results in a significant yield increase over a spring-planted crop [1]. However, freezing temperatures can damage over-wintering plants and significantly reduce their ability to re-grow in spring. Physiological and biochemical adaptation of winter cereals in the fall after planting when temperatures are cold but, above freezing allows them to withstand lower temperatures during winter. This adaptation during fall is called cold-acclimation and has been reviewed extensively [2], [3], [4], [5], [6].

Complicating research on cold acclimation is evidence that certain tissues in the below-ground portion of the stem called the crown, acclimate to a different extent than others [7], [8]. For that reason, it is not uncommon for leaves and roots to die from freezing injury but, during recovery an intact plant grows out of surviving tissues within the crown. In fact, if meristematic tissues survive freezing, then plants will resume normal growth in the spring. Numerous genes have been found to be related to cold acclimation [6], [9], [10], [11], [12], [13]. The expression of some genes in wheat and barley have been localized to the vascular transition zone [14], [15] which is also the region of the crown that is most freezing tolerant [7], [8]. It has been suggested that the expression of other genes are associated with structural changes that occur in crown tissue [8], [16], [17] such as the increase in lignified cells and the development of a barrier between roots and internal crown tissue [18], [19].

In addition to structural changes during cold acclimation, changes after freezing and thawing when plants are placed under growing conditions have also been documented. In an analysis of barley 10 d after freezing, Olien & Marchetti [17] attributed an expansion of injury within the crown to secondary infection from bacteria. In addition to describing extensive freezing damage, they showed images of floral primordia that had developed at the apical meristem in plants that were not severely injured. In wheat, Tannino & McKersie [8] demonstrated significant differences in the vascular transition zone and the apical meristem in the ability to resume growth following freezing. In oat, a Safranin-staining region that appeared to form a barrier separating freeze-damaged from undamaged tissue has been described [16]. Despite these histological analyses only a single report of a 3D analysis of freezing responses has been reported [20].

Many of the changes during recovery from freezing are related to the plants’ ability to repair tissue that has been damaged during freezing [21], [22], [23]. Histological analysis of crown tissue during this period will clarify structural responses of plants to freezing and help physiologists visualize anatomical changes so that recovery from freezing stress can be more clearly understood. In this study we compared the response of the 4 economically important winter cereals, rye, wheat, barley and oats to freezing stress during recovery from freezing. We also document a specific response to freezing found only in oat by digitally reconstructing histological sections in 3 dimensions.

## Materials and Methods

### Plant Growth

Seeds of rye (cv. Rosen), wheat (cv. Jackson), barley (cv. Dictoo) and oat (cv. Wintok) were planted in Fafard 2 Mix (Conrad Fafard, Inc., Agawam, MA in plastic tubes (2.5 cm diameter×16 cm high) with holes at the bottom for drainage, hereafter referred to as “conetainers”. The tubes were suspended in a plastic rack that held 100 conetainers. Plants were treated twice weekly with a modified Hoagland’s nutrient solution [24] and flushed three times weekly with water. Plants were grown for 5 weeks at a day/night temperature regime of 13/10°C with a 12 h photoperiod in a growth chamber with 240 µmol m^−2^ s^−1^ PAR (80% cool white fluorescent and 20% incandescent).

### Cold Acclimation

Five week-old seedlings were transferred to a chamber for cold acclimation (CA) at 3°C with a 12 h photoperiod at 300 µmol m^−2^ s^−1^ for 3 weeks.

### Freeze Tests

The soil surface of conetainers containing CA plants was sprinkled with ice shavings to promote freezing and prevent supercooling. Racks with plants were completely covered with a plastic bag to minimize desiccation during freezing. Plastic bags were loosely sealed and placed into separate freezers (depending on freeze-test temperature), at −3°C. The soil in conetainers, monitored with thermocouples, took approximately 20 h to freeze. After the soil was completely frozen the freezer temperature was lowered 1°C h^−1^ to the following temperatures for each species.

Rye: −12°C, −14°C, −16°C.

Wheat and barley: −10°C, −12°C, −14°C.

Oat: −8°C, −10°C, −12°C.

Each freezer was held at the specific test temperature for 3 h and then gradually warmed to 3°C at a rate of 2°C h^−1^. When the soil temperature reached 0°C racks were removed, allowed to completely thaw under CA (3°C with light) conditions and were moved to growth chambers at 13°C.

### Histology

#### Scanning Electron Microscopy (SEM)

Frozen and non-frozen oat (cv. Wintok) crowns were dissected longitudinally and fixed in 4F:1G (4% formaldehyde, 1% gluteraldehyde in sodium phosphate buffer at pH 7.2) as described by McDowell and Trump [25]. Samples were dried to the critical point in CO_2_, mounted on stubs, and sputter-coated with gold-palladium. Samples were viewed and imaged using a model JEOL JSM-6360LV SEM.

#### Light Microscopy

In the analysis with the four species, plants were removed from conetainers at day 7. For the detailed study involving oat, plants were removed at 0 (right after thawing), 1, 3, 7 and 14 d after freezing in preparation for histological observations. Plants were rinsed in water and the bottom 1 cm of the crown was placed in FAA fixative (modified with methanol [26]). Crowns from unfrozen plants, grown identically to the frozen plants, were also collected at each time period to use as control samples.

Crowns from each treatment were subjected to microwave fixation, dehydration, and paraffin embedding process described previously [27]. For the initial experiment, 4 frozen and 4 unfrozen crowns from each species were processed. For the detailed experiment involving oat, 7 crowns from each recovery period as well as unfrozen controls were used. In the experiment with the four species each crown was sectioned at 25 µm longitudinally in a rotary microtome. In the detailed analysis of oat each crown was cut in cross section from the bottom of the crown to just above the primary apical meristem.

Crown sections were stained in Safranin for 2 min, rinsed in water and then in 50% ethanol. They were then placed in fast green for exactly 1 min and rinsed in ethanol, and then xylene and covered. This staining treatment prevented nuclei from staining red; the only areas of the tissue to stain red were regions showing a response to freezing and a small amount of dead, presumably lignified tissue. This stain protocol permitted a consistent and repeatable colorimetric assay to quantify the freezing response within each section.

Sections were photographed at 40X with a digital camera (Sony DSC707) attached to a Nikon Eclipse 50 i microscope and illuminated with a 30 W halogen lamp. To quantify staining intensity in the detailed analysis, JPEG images were imported into SigmaScan Pro (version 5.0. Systat Software Inc, San Jose CA). First, a color range that encompassed all of the tissue in each image, but none of the background, was selected so an accurate measure of the volume of each crown could be determined. Then the color of several images of frozen plants was compared to that of unfrozen plants and a color range that encompassed only the visible reddish barrier was selected so that this particular freezing response could be quantified. A macro was then used to analyze all the images (from 100 to 300 images depending on the height from the bottom of the crown to the top of the apical meristem) for each sample.

The number of pixels for each color determined by SigmaScan was converted to mm^2^ and the area of color for each image was totaled for each crown. Each crown was considered to be a replication. Seven replications were used to obtain the average of the colors in each treatment.

A Nikon BV-2B filter set was used to photograph auto-fluorescence. This filter set excites between 405 and 445 nm with a dichromatic mirror cut-on wavelength of 460 nm and a barrier filter cut-on at 475 nm.

### Tetrazolium Analysis

Oat crowns recovering at the time periods listed above were cut longitudinally with a razor and incubated with 0.5% 2,3,5 triphenyl tetrazolium chloride in a 50 mM HEPES (pH 7.3) solution for 24 h at 21°C. Unfrozen controls were incubated in an identical solution. The half crowns were photographed under a dissecting microscope at 12X with surface lighting.

### 3D Reconstruction

From 150 to 300 JPEG images were imported into After Effects (Adobe Systems Inc, San Jose, CA). Images were aligned manually and color-keying was used to remove the background from all the images simultaneously. The same color-keying effect was then used to remove the color of green staining tissue, leaving only the red color for all the images. This "digital clearing" allowed us to observe inside the tissue at any object that stained differentially. Images were then distributed evenly in the 3^rd^ dimension using a plug-in called AlignLayers (freeware available on line).

Once the images were aligned and the background was made transparent, a 3D composition was created with a “null” layer that allowed simultaneous manipulation of all the images as a 3D volume. The null layer was then animated to rotate the 3D object around all three axes (see Videos S1 and S2). See Livingston, et al., [20] for more details on the procedure used.

## Results and Discussion

### Differences and Similarities between the Histology of Rye, Wheat, Barley and Oat: Unfrozen Controls

**Figure 1:**
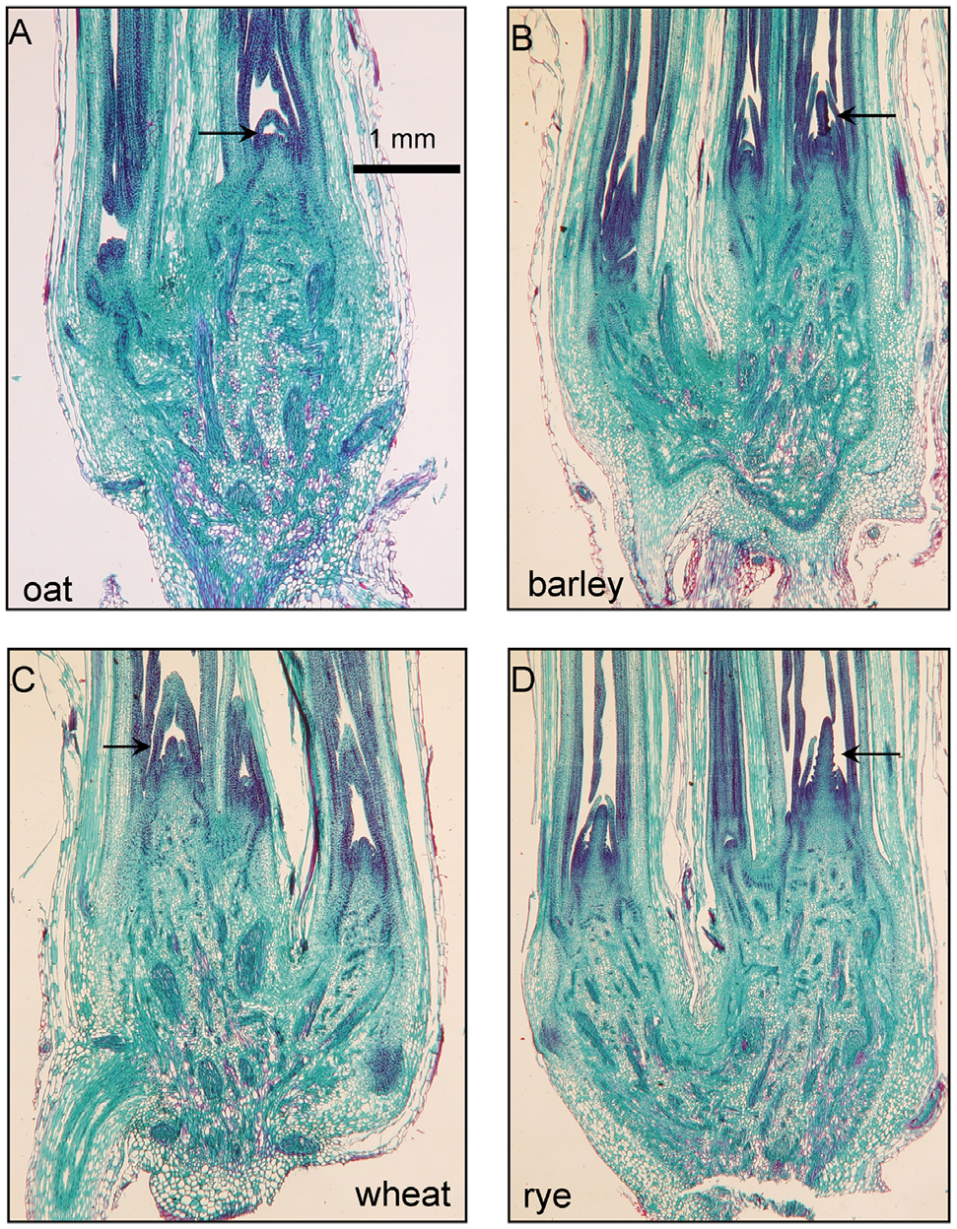
Longitudinal sections of non-frozen (control) winter cereal crowns. Arrows in each image indicate the position of the shoot apex of the primary tiller of each species.

Crowns of all four species (Fig. 1) in their cold acclimated and unfrozen condition had a similar internal structure to oats as described previously [16]. All four species had multiple stems or tillers, each with its own shoot apex and vascular bundles scattered throughout the crown in a seemingly random fashion. There were generally four overlapping zones within the crown in all the species. The four zones starting at the bottom of the crown were designated [27] as: the lower crown, crown core, transition zone and shoot apex. In a previous study [27] comparing frozen to unfrozen oat crowns, the lower crown had significantly higher concentrations of fructose, glucose and fructan but significantly lower concentrations of sucrose, than the other three zones.

The only obvious visible difference between the four species (Fig. 1) was in the shoot apex of the primary tiller. The shoot apex of oat and wheat had a similar shape and was generally shorter than either barley or rye. Both rye and barley had a longer shoot apex but in rye the shape was more triangular than in barley. Aloni and Griffith [18] in a histological comparison of these same four species (as well as maize, sorghum and corngrass) reported that rye had the highest degree of vascular segmentation in the crown. This reportedly helps restrict ice growth into the crown [18] and may be one reason rye is the hardiest of the four winter cereals.

### Differences and Similarities in Frozen Crowns of Four Winter Cereal Species

**Figure 2:**
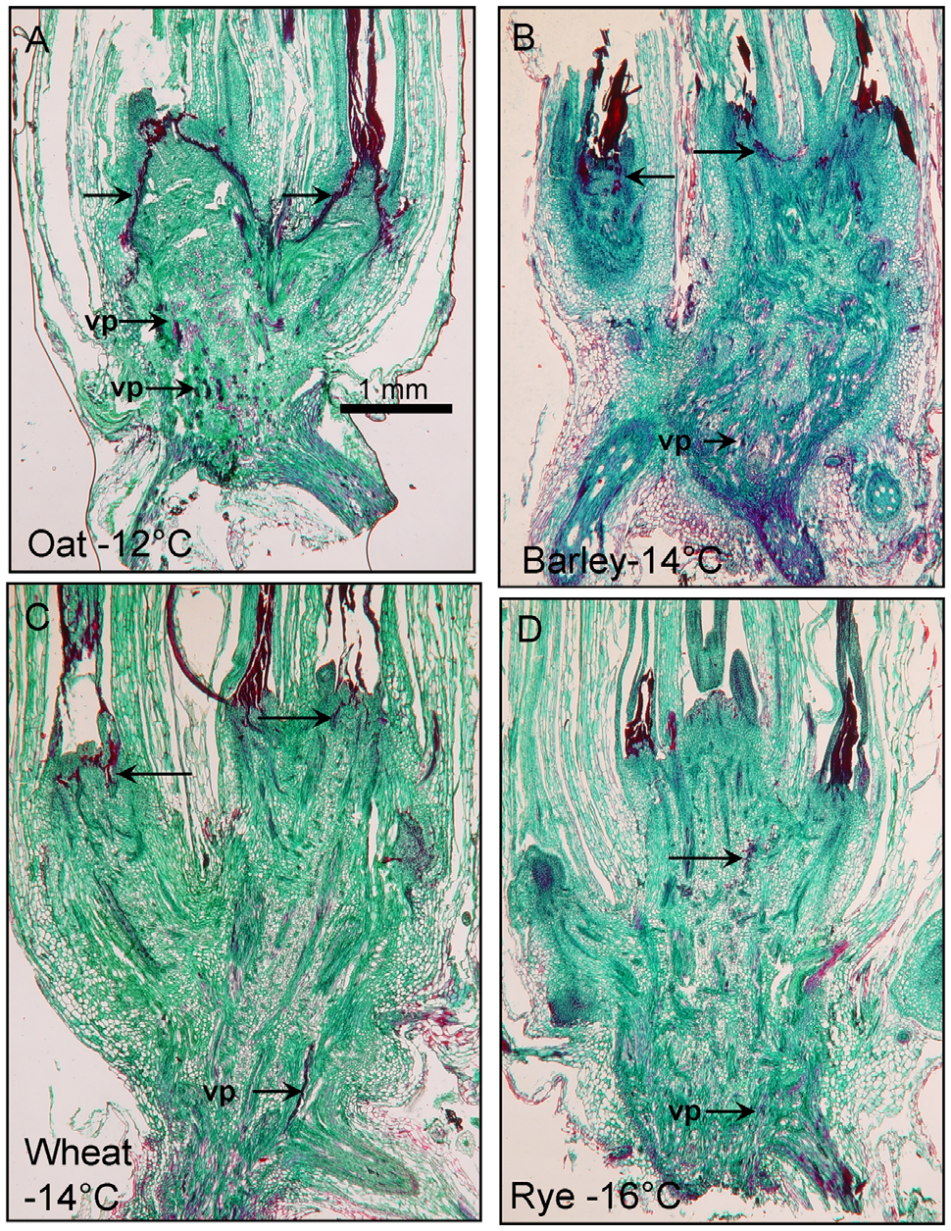
Longitudinal sections of winter cereal crowns 7 days after freezing and thawing. Each species was frozen at its respective LT50 which is indicated at the bottom of each panel. Note the severely distorted shoot apexes of each species as compared to the unfrozen apexes in Fig. 1. Unlabeled arrows indicate regions of the crown with barrier-like freezing responses. Note the continuous barrier in oat that was not seen in the other species. “vp” signifies areas of vessel plugging in the lower crown.

The primary difference between the four species when they were frozen at warmer temperatures (not shown) was that shoot apices in all four species were more likely to survive. At colder temperatures, freeze-damage was simply more wide-spread within the crown. Images included here (Fig. 2) are typical of differences and similarities in freeze-response among the species.

All four species had one or more shoot apices that had been severely damaged by freezing as evidenced by dark red undifferentiated tissue where the shoot apex had been (Fig. 2; compare to shoot apices of unfrozen controls in Fig. 1). Safranin stains lignified, cutinized and suberized tissue a deep red [26], [28], [29], [30] and under some counter-staining protocols nuclei also retain the red color [26]. While the red staining regions in figure 2 may suggest an increase in lignin compounds in frozen tissue, more analyses will be needed to confirm this. Another common visible freeze-response was apparent plugging of vessels with dark red-staining material in the lower crown (Fig. 2, arrows); this response was more common in oat and least common in rye. This plugging was previously observed in oat and was attributed to bacterial necrosis or possibly coagulated protein in damaged xylem vessels [16]. Somewhat narrow regions of red-staining tissue of varying lengths were also commonly seen throughout the upper part of the crown in frozen samples (Fig. 2). While not always the case, these red staining regions seemed to divide undamaged tissue from that which had been damaged by freezing. This red-staining region was always more pronounced in oat. Regardless of the extent of injury, the response was not seen to the same extent in rye, wheat or barley (Fig. 2). It was visible at all three freeze temperatures in oat (−8, −10 and −12°C) with the only difference between temperatures being: at colder temperatures tissue disruption pushed through the transition zone and severely distorted the shoot apex (not shown). The presence of this unique freeze-response in oat led us to characterize it histologically in more detail (below).

### Tetrazolium Analysis Delineating Live and Dead Tissue in Frozen oat Crowns

**Figure 3:**
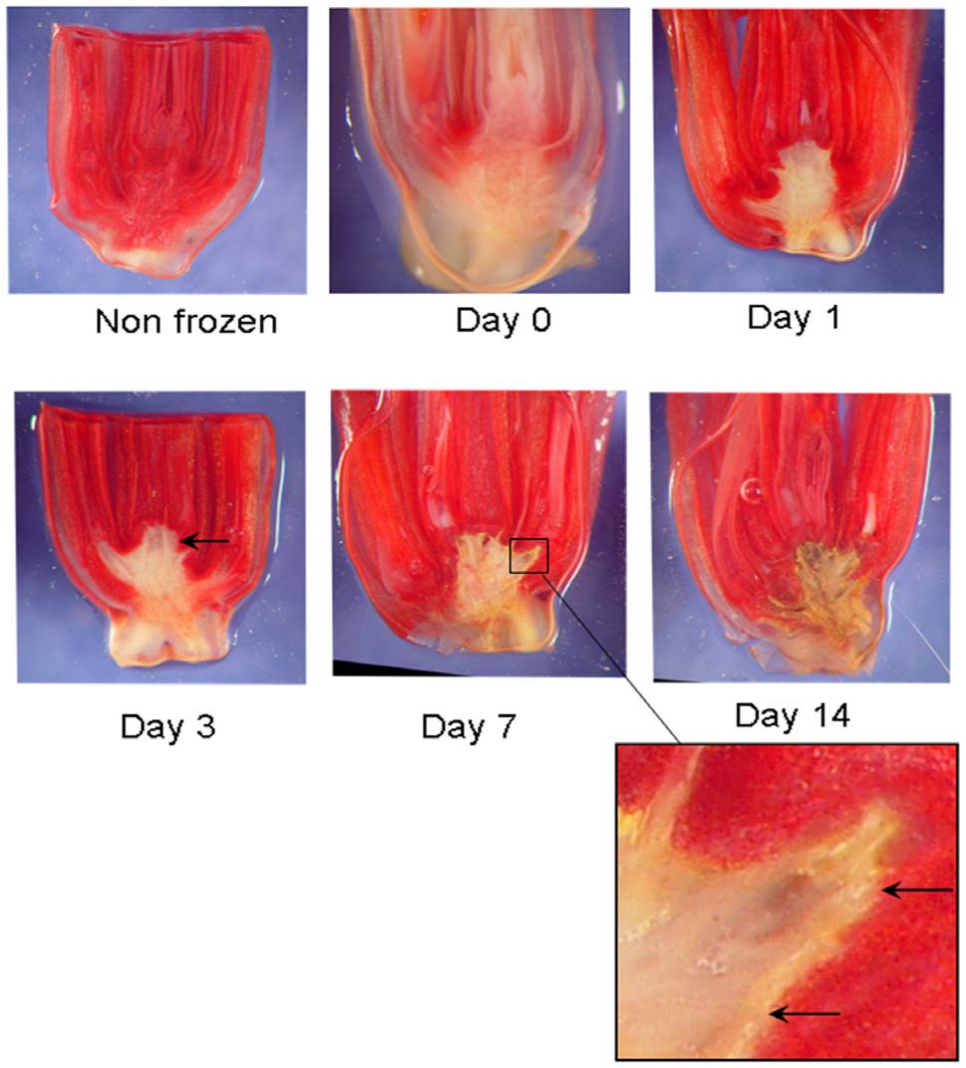
Hand sectioned oat crowns stained with tetrazolium. A non-frozen control crown is compared to crowns sectioned at various time periods after freezing and thawing. A red color identifies tissue with the ability to reduce tetrazolium, indicating live cells while yellow to white tissue is considered to be dead as a result of freezing. Arrows at day 3 and day 7 (inset) show where the barrier separates live from dead tissue. Note the dramatic change in tetrazolium reduction between day 0 and day 1.

The red, Safranin-staining region formed a layer in longitudinal sections that surrounded the upper and central part of the crown (Fig. 2). This layer has been called a barrier because it appeared to separate living from dead tissue [16]. In this study we confirmed the separation of living from dead tissue by tetrazolium staining in fresh, hand-sectioned crowns (Fig. 3). Tetrazolium is reportedly reduced by electrons from the electron transport system of the mitochondria [31] forming a red-colored derivative, formazan; this allows the visual distinction of live (red) from dead (white) cells. It is used routinely to test the viability of stored seed [32]. It has also been used as an indicator of vegetative cells which survived freezing [8], [28].

One day after freezing, the dead zone, as visualized by the lack of tetrazolium staining (white) in the center of the crown, was smaller than it was just after freezing at day 0 (Fig. 3). Since tetratzolium is reduced by electrons from the mitochondria [31] this suggested possible mitochondrial damage during freezing. As early as day 1, much of the tissue within the crown was able to reduce tetrazolium suggesting a recovery of at least some of the cells within the crown. By day 3, even before the barrier was fully visible with Safranin (as it was in day 7), a clear demarcation in the center of the fresh crown was visible between live and dead regions. When the barrier had become fully visible at day 7, tetrazolium reduction occurred up to but not within the barrier (Fig. 3, see inset at day 7). By day 14, tissue within the barrier was dark yellow and showed signs of significant degeneration, even though tissue was alive just outside the barrier.

**Figure 4:**
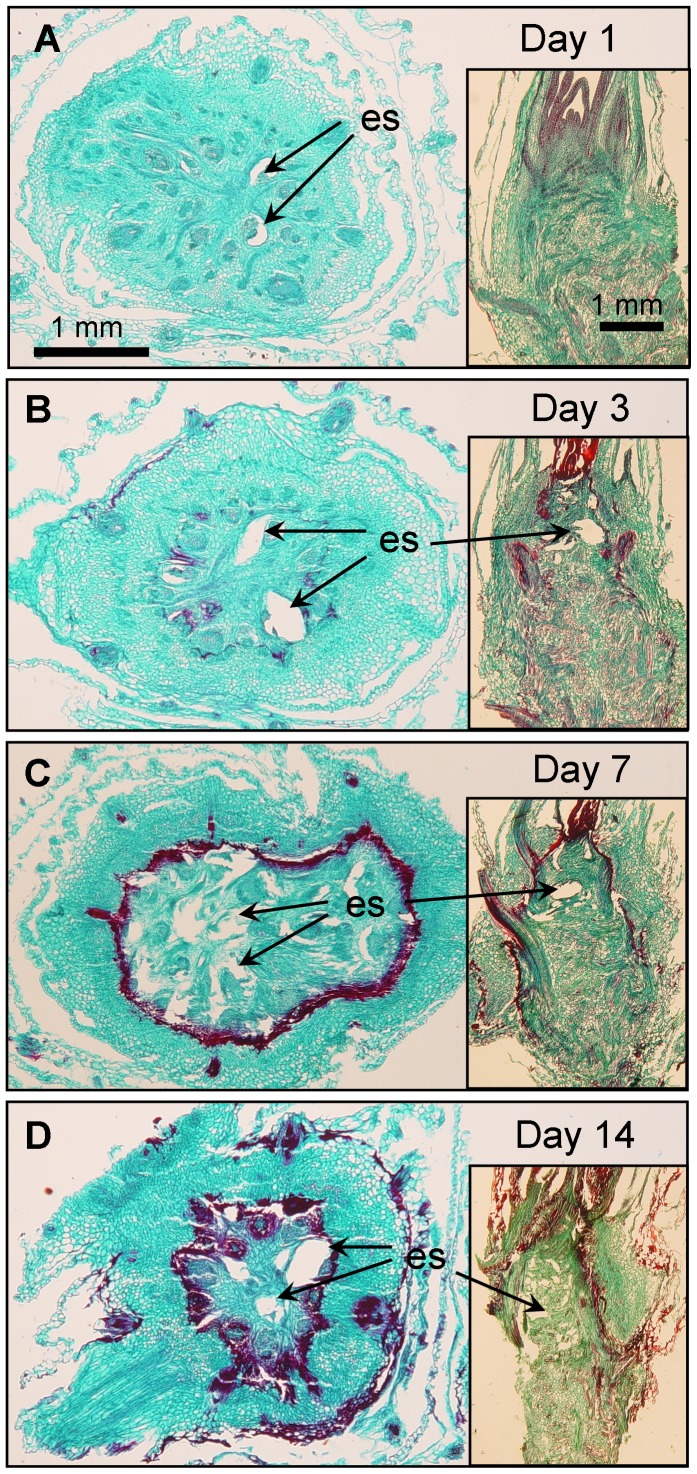
Cross sections of oat crowns at 4 time periods after freezing and thawing. The inset image in each panel is a longitudinal view of the crown at the same time period. ES = empty spaces caused by ice disruption and tissue degeneration. A) Note that no barrier is visible 1 day after freezing and thawing and B) by day 3 the barrier is beginning to develop. C) The barrier is fully developed by day 7 forming an out of shape circle which appears to confine the empty spaces. D) The red staining area outside the barrier at day 14 is dried epidermal tissue on the stem.

Visible damage inside the barrier consisted of pycnotic nuclei (not shown here, see [16]), disintegrated cells and empty regions of disrupted tissue (Fig. 2, Oat). Some of the empty regions appeared to be caused by the growth of ice crystals during freezing because even at day one, before the barrier was apparent, empty spaces were visible (Fig. 4). It is likely that expansion of the empty spaces during recovery were simply caused by degeneration of freeze-damaged tissue.

It should be noted that while empty spaces between leaf whorls were seen outside the barrier, freeze-induced tissue disruption, was only observed inside the barrier even before the barrier became fully visible at day 7 (Fig. 4). It is possible that those cells giving rise to the barrier restricted ice crystal growth much the same way the root-shoot barrier described by Aloni and Griffith [18] in rye, barley and wheat.

**Figure 5:**
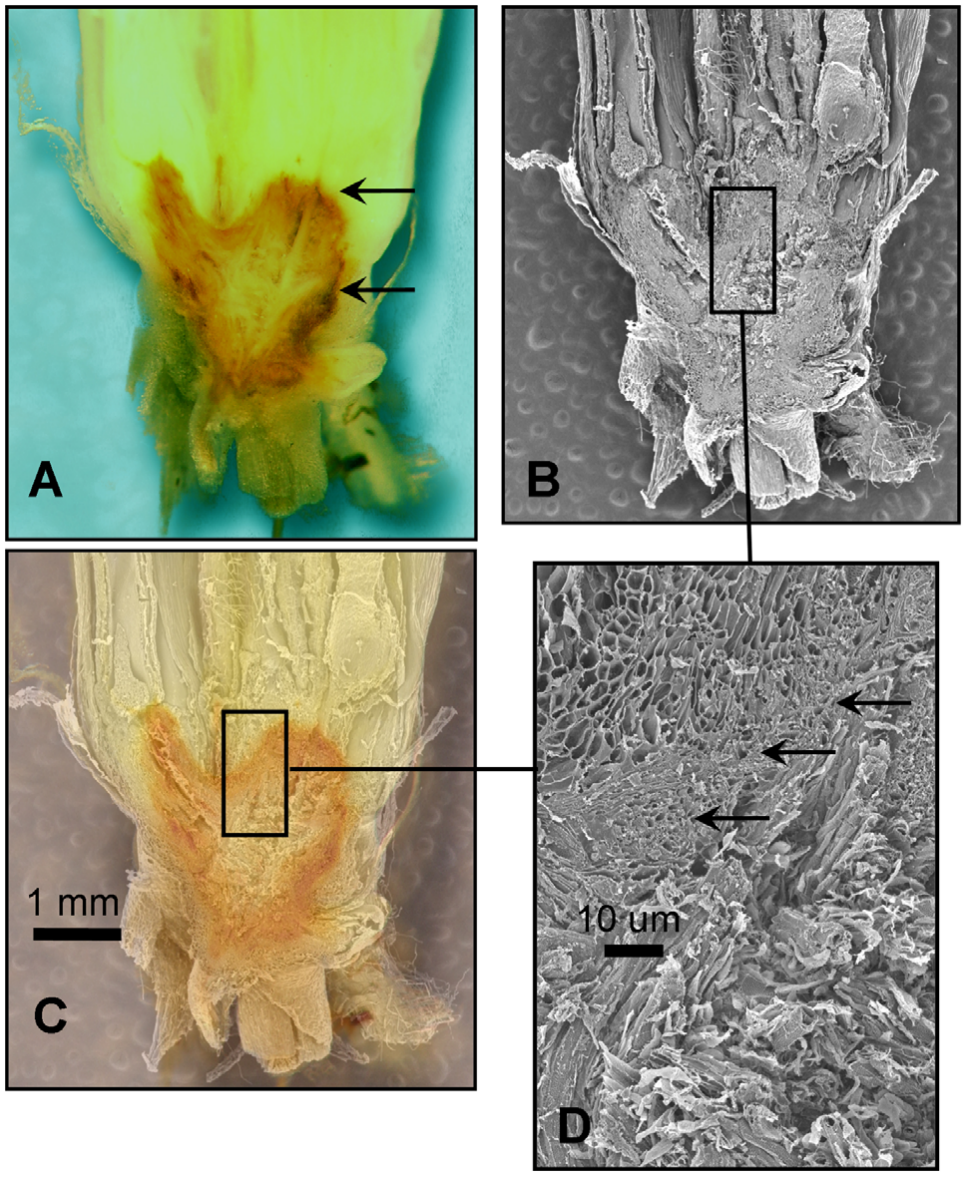
Hand section of a fresh (unfixed, unstained) oat crown 7 days after freezing. A) Note the outside edge of the barrier (arrows). B) the other half of the crown visualized by scanning electron microscopy (SEM). C) SEM image overlaid with the fresh section in A. D) a closer view of part of the barrier (arrows) under SEM. Note the relatively compact cells within the barrier.

Scanning electron microscopy (SEM) of the same region on the other half of the crown indicated that the barrier was within a region of cells that were more tightly packed than those outside the barrier (Fig. 5). Aloni and Griffith [18] state that the tracheary elements at the root-shoot junction that are responsible for the barrier are “small cells with simple wall pitting”. While simple wall pitting was not observed, the barrier producing cells (Fig. 5) appeared considerably smaller than cells within and without the barrier. Inside the barrier of the frozen crown, SEM showed a distinctly disrupted appearance in contrast to the cells outside the barrier (Fig. 5).

**Figure 6:**
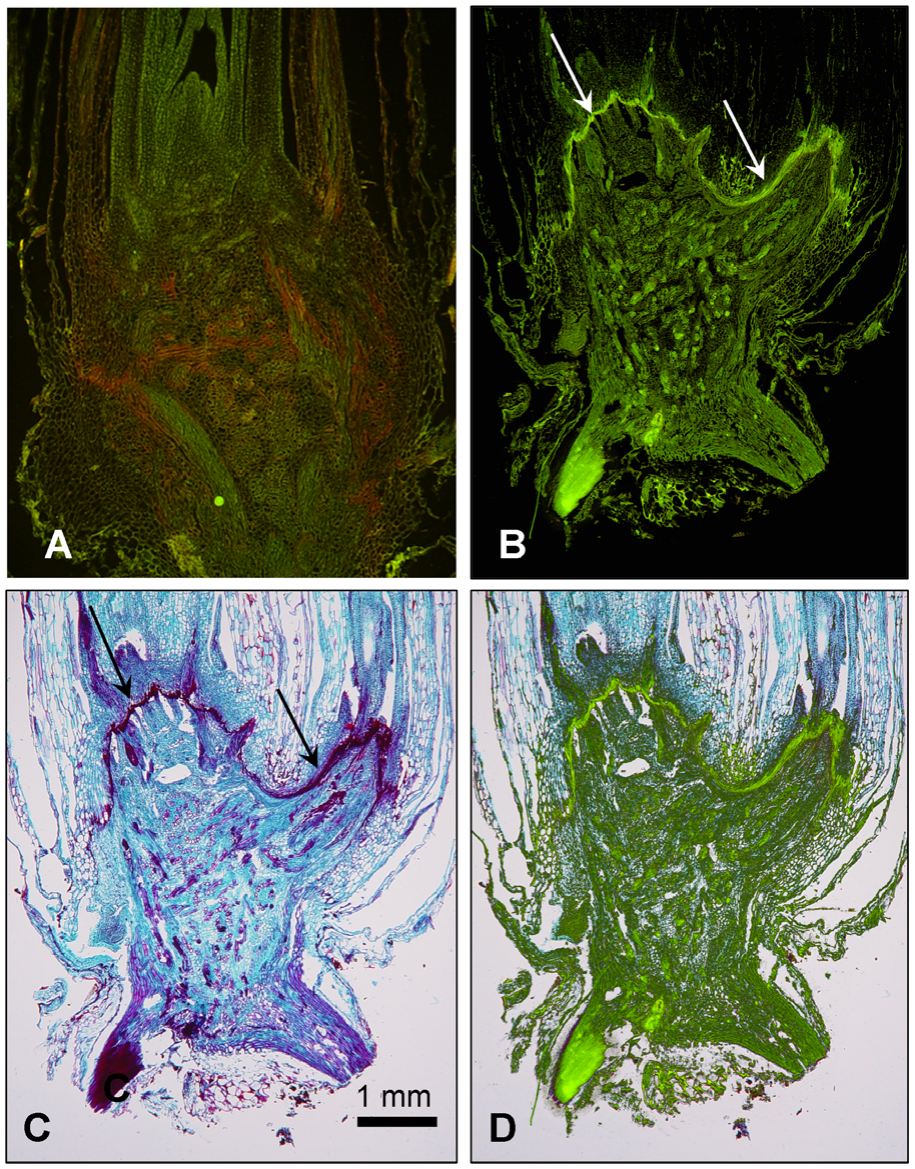
Auto-fluorescence of longitudinal sections of unstained oat of crowns. A) Non-frozen control. B) 7 days after freezing at −12°C. C) Safranin, fast-green stained section of A after imaging autofluorescence. Note the intense fluorescence of the barrier (arrows) that is not present in the unfrozen section in A. D) Autoflouresence image B overlaid with the Safranin-stained image C.

The autoflouresence of the barrier (Fig. 6) suggested that it may be composed of lignins and/or phenolics since most of these are strongly autoflourescing [33]. Kitin et al. [33] state that plants are capable of regulating lignin compounds in response to environmental changes and localize their presence in stems of poplar trees to xylem vessels using cryo-flouresence microscopy.

In a preliminary HPLC analysis to detect fluorescing lipid and polar metabolites there was a clear increase in the number and quantity of fluorescing compounds observed in the frozen crowns (not shown) consistent with the observation of a layer of fluorescent tissue surrounding the dead tissue in the frozen crowns (Fig. 6). A detailed investigation is underway to determine the identity of the compounds and to understand how they might be related to the recovery process.

### Quantitative Color Analysis of the Internal Freeze-response Demonstrating an Increase Over Time after Freezing

**Figure 7:**
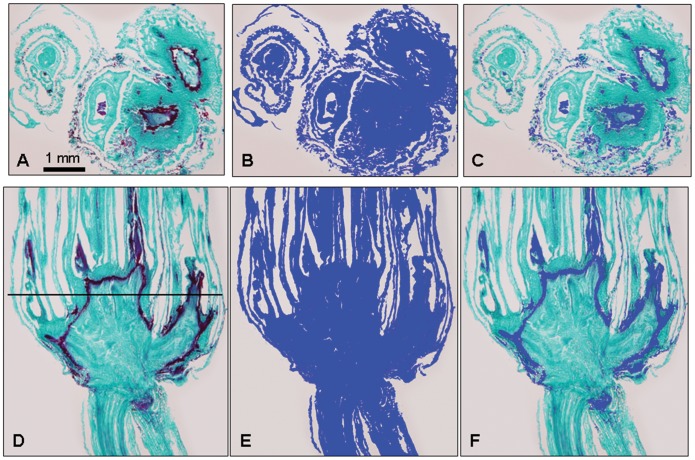
Stained oat crown 7 days after freezing and thawing illustrating color recognition by SigmaScan. The blue color identifies pixels that SigmaScan recognized. A) original image before color processing. B) SigmaScan differentiating crown tissue from background. C) SigmaScan recognizing the barrier as distinct from the rest of the tissue. D) to F) longitudinal section of a different crown treated as in A. The bar in D represents the approximate position that the cross section was taken in A. Percentages shown in Fig. 8 are the pixel counts of the barrier in C divided by the total tissue in B.

**Figure 8:**
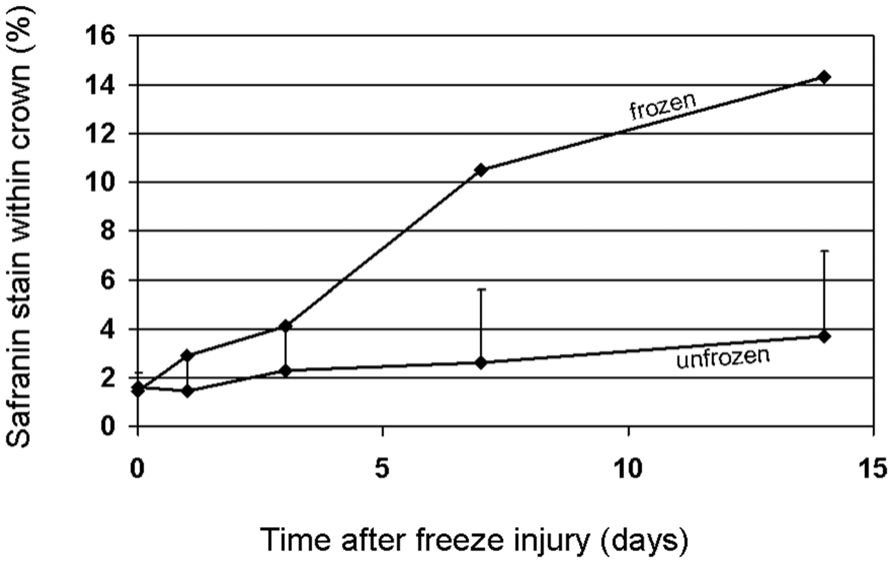
Change over time after freezing in the percentage of red color due to Safranin staining within oat crowns. Each data point is the mean of 7 plants and each plant is the sum of the red color in 120 to 180 sections, depending on the height of the crown. The bars on the unfrozen control line show the least significant difference (Tukeys HSD) at P≤0.05).

Although many histological studies have described freeze-induced changes in crown tissues, techniques to quantitatively document that damage are limited. By modifying an established Safranin staining protocol (see materials and methods) we were able to prevent nuclei from staining red and effectively minimize red staining cells in unfrozen tissue. This meant that tissues staining red in frozen crowns could be regarded as essentially a freeze-response since those tissues only stained red in plants that had been frozen. This allowed color recognition software to selectively quantify red colored pixels within individual sections of crown tissue (Fig. 7) and thus provided a quantitative measurement of freeze-response (Fig. 8).

In both frozen and unfrozen plants 2% of the total volume of the crown stained red with Safranin at day zero (Fig. 8). Safranin typically stains lignified tissue and in both frozen and unfrozen crowns there is a small amount of lignified leaf tissue on the outside of the plants; this increased slightly to 3% in both treatments from day zero to day 3. In frozen plants the increase was due primarily to the faint outline of barrier that seemed to originate from a ring of cells surrounding the crown core (Fig. 4). During the time course following freezing, the barrier appeared to remain in the same position in the crown, but became more prominent (Fig. 4). After day 1 the percentage of red color in frozen crowns increased significantly more than in unfrozen tillers. By day 14, a little over 14% of the total volume of frozen crowns had stained red. A small portion of the red stain that was quantified at day 14 consisted of dead epidermal tissue outside the barrier (Fig. 4). Unfrozen crowns remained at or just above 2% for the entire time course (Fig. 8).

### 3D Reconstruction Demonstrating Anatomical Continuity of the Barrier

**Figure 9:**
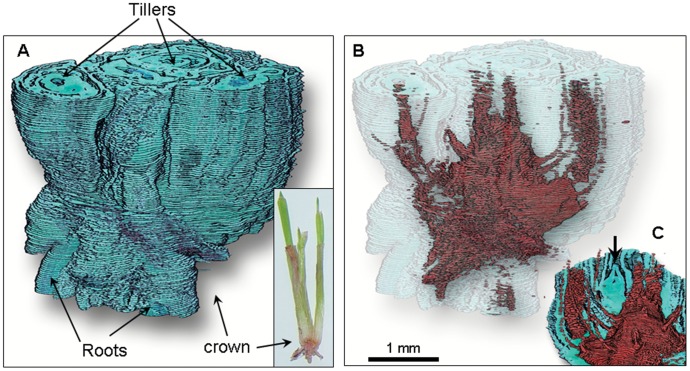
3D reconstruction of an oat crown 7 days after freezing. A) Reconstruction of 126 sequential sections that were aligned and distributed in z-space. B) The same crown in A but with green tissue partially cleared to reveal barrier (false color) and dead apical meristems inside. C) a digital cross section of the primary and secondary tiller from a different angle, showing a single live meristematic bud (arrow). See Video S1 for a clearer 3D view.

**Figure 10:**
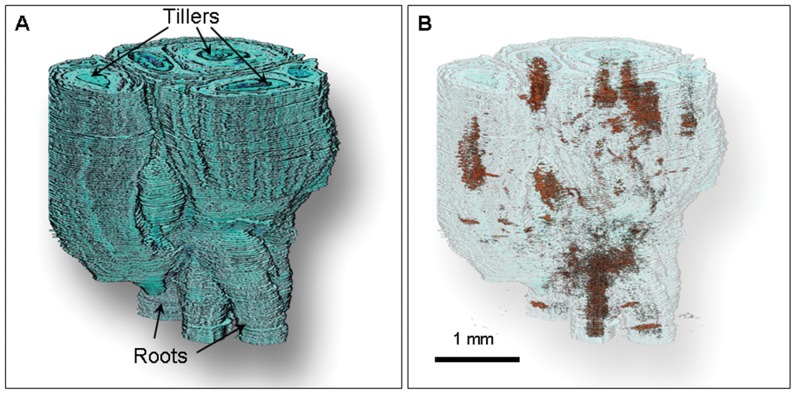
3D reconstruction of an oat crown 3 days after freezing. A) Reconstruction of 155 sequential sections that were aligned and distributed in z-space. B) The same crown in A but with green tissue partially cleared to reveal initial formation of the barrier. See Video S2 for a clearer 3D view.

When images of multiple sections were aligned, distributed in z-space and background color was removed, a 3D reconstruction of the crown was created (Fig. 9, Video S1). Using a digital clearing technique described previously [20] we were able to decrease the opacity of surrounding tissue and visualize the barrier within the crown in 3D. At day 3, with the barrier faintly visible, the red staining regions appeared to be scattered throughout the crown core (Fig. 10 and Video S2). By day 7 the barrier had fully developed and formed a roughly spherical shape (Video S1) with what looked like openings (regions with no red) where root tissue entered into the crown (visible in the supplemental video). The red color extended upwards into individual tillers where apical meristems were considerably distorted. A single apical bud which had either survived freezing or had been initiated after the apical meristem was distorted by freezing, remained green with no red staining (Fig. 9). On day 14 the 3D reconstruction (not shown) was similar to that at day 7.

This 3D reconstruction technique is ideally suited for *in situ* hybridization studies with compounds that may be expressed either during cold acclimation or freezing or recovery from freezing. *In situ* hybridization in combination with 3D reconstruction would anchor metabolites to specific tissue which in turn would allow visualizing the precise location of the metabolites in 3D space. The current method of grinding plant organs and extracting metabolites provides very little if any information as to the location of those metabolites within live and/or dead tissue. The precise location of gene expression in the plant is important since there are clearly defined regions of live versus dead tissue (Fig. 2) with presumably different gene expression in each region.

Though the barrier seems to be a response to freezing, its specific function is unknown. Because Safranin stains primarily lignified tissue, we suspect that the putative barrier is a form of lignin that was released from phenol producing cells and diffused into surrounding tissue [34]. The autoflourescence of the barrier (Fig. 6) supports the hypothesis that it is phenolic in nature. Beckman [34] states that phenols can stabilize regions of tissue and “in the process, create of the cells one large, durable inert macromolecule”. The phenol-producing cells Beckman describes are stimulated by pathogen infection, while we observed the barrier in plants that had been frozen. It is possible that the barrier-producing cells in this study are reacting to secondary infection since it has been shown that post-freeze infection by *Pseudomonas* bacteria is common [35], [36]. In fact, Olien and Smith [35] found *Pseudomonas* moved from the roots of barley into crown tissues after freezing and they attributed a portion of the tissue death within frozen crowns to bacterial as well as fungal infection.

While the barrier was observed in a limited number of freezing tolerant oat cultivars (a second freezing tolerant oat cultivar, Norline, also had a prominent barrier (not shown)), we have never observed it in the most freezing tolerant winter cereal, rye, even when frozen to the point (−16°C) where apical meristems were severely distorted and presumably killed (Fig. 2). In a preliminary tetrazolium analysis of frozen wheat crowns recovering from freezing, we saw a distinct demarcation of live from dead tissues (not shown), similar to what was found in oat (Fig. 3) but this region did not stain with Safranin as it did in oat (Fig. 2). This suggests that a barrier may also exist in wheat as was suggested by Tanino and Mckersie [8]. However, the barrier in wheat is likely of a different composition than oat since it did not stain in the same way (see Fig. 2). If the barrier as seen in oat is a form of protection to limit cell/tissue death after freezing, it is also possible that rye, barley or wheat do not need this mechanism for recovery because they would likely be able to withstand the freezing stresses causing the initial injury.

### Conclusion

Winterhardiness is arguably the most complex issue related to successful cultivation of winter cereals. Numerous reports on cold and freeze acclimation have described various freezing survival mechanisms. One aspect of winterhardiness that has received little attention is the period of recovery following freezing when repair mechanisms could significantly impact how, or if, a plant survives freezing. As a follow-up to a previous study [20] we have presented here a more detailed 3D reconstruction of the internal response of crown tissue during a recovery period following freezing. While it is doubtful that one or two simply inherited traits will explain this important aspect of winter survival, research involving post-freeze recovery will provide a more comprehensive understanding of overall winterhardiness.

## Supporting Information

Video S1 **Video of 3D reconstruction of an oat crown 7 days after freezing (see text for** **Figure 9** **).**
(MP4)

Video S2 **Video of 3D reconstruction of an oat crown 3 days after freezing (see text for** **Figure 10** **).**
(MP4)
